# Low-Salt Intake Suggestions in Hypertensive Patients Do not Jeopardize Urinary Iodine Excretion

**DOI:** 10.3390/nu10101548

**Published:** 2018-10-19

**Authors:** Natale Musso, Lucia Conte, Beatrice Carloni, Claudia Campana, Maria C. Chiusano, Massimo Giusti

**Affiliations:** Centre for Secondary Hypertension, Unit of Clinical Endocrinology, Department of Internal Medicine, University of Genoa Medical School, IRCCS Ospedale Policlinico San Martino, 16132 Genova, Italy; luciaconte88@hotmail.it (L.C.); beatrice.carloni@gmail.com (B.C.); claudiadindon@hotmail.it (C.C.); mcristinachiusano@libero.it (M.C.C.); magius@unige.it (M.G.)

**Keywords:** blood pressure, dietary sodium, hypertension, iodine, salt

## Abstract

A low-sodium diet is an essential part of the treatment of hypertension. However, some concerns have been raised with regard to the possible reduction of iodine intake during salt restriction. We obtained 24-h urine collections for the evaluation of iodine (UIE) and sodium excretion (UNaV) from 136 hypertensive patients, before and after 9 ± 1 weeks of a simple low-sodium diet. Body mass index (BMI), blood pressure (BP), and drug consumption (DDD) were recorded. Data are average ± SEM. Age was 63.6 ± 1.09 year. BMI was 25.86 ± 0.40 kg/m^2^ before the diet and 25.38 ± 0.37 kg/m^2^ after the diet (*p* < 0.05). UNaV decreased from 150.3 ± 4.01 mEq/24-h to 122.8 ± 3.92 mEq/24-h (*p* < 0.001); UIE decreased from 186.1 ± 7.95 µg/24-h to 175.0 ± 7.74 µg/24-h (*p* = NS); both systolic and diastolic BP values decreased (by 6.15 ± 1.32 mmHg and by 3.75 ± 0.84 mmHg, respectively, *p* < 0.001); DDD decreased (ΔDDD 0.29 ± 0.06, *p* < 0.05). UNaV and UIE were related both before (*r* = 0.246, *p* = 0.0040) and after the diet (*r* = 0.238, *p* = 0.0050). UNaV and UIE were significantly associated both before and after the diet (*p* < 0.0001 for both). After salt restriction UIE showed a non-significant decrease remaining in an adequate range. Our dietary suggestions were aimed at avoiding preserved foods, whereas the cautious use of table salt was permitted, an approach which seems safe in terms of iodine intake.

## 1. Introduction

A low-salt diet constitutes a standard approach to the treatment of hypertensive patients [1,2,3,4]. Salt is the fundamental source of dietary sodium, an excess of which is linked to hypertension and cardiovascular diseases [5,6,7]. While reducing excess dietary sodium is universally acknowledged to be a favorable step in the reduction of cardiovascular risk [1,2,3,4], the recommendation of a low-sodium approach in the general population is still controversial [8]. Recently, a J-shaped curve linking sodium intake to cardiovascular events has been proposed [9,10], pointing to a possible risk induced by a decreased sodium intake in the general population. However, these data have been disputed [11].

In Italy iodine supplementation was first undertaken in 1921 and iodized salt was introduced by regulatory authorities in 1924 [12]. Since then, substantial progress has been made in the struggle against iodine deficiency [13]. Nevertheless a mild insufficiency persists [14] and only recently (Law n.55/2005) was a nationwide salt iodization program implemented [15].

The main concern raised by the reduction of sodium intake regards the possible deficiency of iodine intake during a low-sodium diet [16]. In hypertensive patients in whom iodine status (IS) has been evaluated by 24-h urinary iodine excretion (UIE) or by spot urine iodine concentration (UIC), or by anamnestic instruments such as 24-h dietary recalls or food frequency questionnaires (FFQ), no general consensus has emerged [17,18]. This may partly stem from the different methods employed, the greatest limitation being associated with anamnestic evaluation [19] and with the variability of spot urine measurements versus the gold standard of UIE [20,21]. Another issue concerns the general approach of studies in which sodium and iodine intake have been evaluated, in accordance with an observational protocol, whereby it is assumed that patients displaying a reduction in urinary sodium excretion are following a low-sodium diet, while those displaying no reduction are not [22,23]. Few intervention studies have shown adequate levels of iodine intake after salt restrictions [24,25].

An additional problem arises from the type of population examined. As a general rule, when an iodine-sufficient population is studied, patients on a low-sodium diet show reduced UIC/UIE, but no substantial change in IS [26]. When iodine-deficient patients follow a low-sodium diet, IS worsens [17,27].

In Italy, these concerns have already been raised [28]. Iodine deficiency leads to endemic goiter and cretinism, because iodine is essential for the synthesis of thyroid hormones. In our country public committees were appointed since the first half of the XIX century, for the identification of areas of endemic goiter and endemic cretinism, mostly in the north-western and insular areas [15]. Since then, many epidemiological evidences showed that iodine deficiency is present in mountain as well as in coastal regions [15]. Although our district, the Liguria Region, is situated in a country of mild iodine deficiency, it has recently become iodine-sufficient [29].

The aim of the present study was to evaluate the modification of UIE induced by a proposed low-sodium diet in hypertensive patients. Urinary iodine excretion and 24-h urinary sodium excretion (UNaV) were measured by standard methods before and after the administration of a dietary protocol.

## 2. Materials and Methods

In a larger cohort of 291 hypertensive patients [30], we obtained a 24-h urine collection (above at least 700 mL) from 157 patients for UIE and UNaV evaluation (basal value: time t0), together with BMI and blood pressure (BP) measurements. A low-sodium diet was then proposed. After 9 ± 1 weeks (mean ± SD) we repeated the 24-h urine collection (time t2), BMI recording, and BP measurements.

The low-sodium diet prescribed by a dietitian was based on simple recommendations printed on a single A4 sheet of paper [30]. Patients were advised to avoid salty foods, ice-cream, cheese, and cured meats, such as bacon, ham, sausages, and so on. Low-sodium bottled water was recommended. The patients were also asked to switch from regular bread to salt-free bread, which is commonly available in Italy. Table salt itself was not banned, although limited use was suggested [30].

Twenty-four hour urines were collected in accordance with a standard protocol: after the first morning void (to be discarded) patients were requested to record the time and to collect every subsequent void until the same time the following day, when they had to collect the content of the last void. All patients received written and verbal instructions, together with the appropriate urine containers.

Blood pressure was measured by means of a semi-automated repeated-measures method (HEM 907 BP monitor, OMRON, Kyoto, Japan) as previously described [30] both at t0 and at t2. Blood pressure was measured three times at each visit and the last value (BP3) was considered for analysis. Body weight and BMI were obtained both at t0 and at t2. Anti-hypertensive drugs (as defined daily doses-DDD following the WHO definitions) [31] were recorded both at t0 and at t2. Halfway between the two visits, patients were suggested to have their BP evaluated by their general practitioner (who was aware of the study but blind to the results), which adjusted their drug treatment, if necessary [30].

UNaV was measured by an AutoAnalyzer (COBAS 8000 Roche/Hitachi with an ISE module; Roche Diagnostics, Indianapolis, IN, USA), while urinary iodine was measured by means of a current commercial colorimetric method (Celltech, Turin, Italy) all CVs are below 8%.

All patients were on anti-hypertensive drug treatment. Patients on thyroid hormones, amiodarone or with a recent history of contrast-media exposure were excluded. Patients unable or unwilling to provide two (t0 and t2) 24-h urine collections (i.e., those with less than 700 mL of urines and/or violation of void collection), or with concomitant diseases such as congestive heart failure, atrial fibrillation, renal failure, diabetes, electrolyte abnormalities, secondary hypertension, or goiter were deemed ineligible.

Finally, 136 patients, who had successfully provided 24-h urine collections twice in a two months period, were selected. Neither FFQ nor 24-h dietary recalls were used. The study population consisted of Caucasian Europeans, 98%, and Latin Americans, 2%. Of the 136 patients, 84 (61.76%) claimed to use iodized table salt routinely, in agreement with our national data [15]. All subjects gave their informed consent for inclusion before they participated in the study. The study was conducted in accordance with the Declaration of Helsinki, and the protocol was approved by the Ethics Committee of our Hospital.

### Statistics

Statistical analysis was performed using a commercial software package: PRISM 7.0 (Graph Pad Software, La Jolla, CA, USA). Paired Student’s *t*-test, non-parametric analysis (Mann-Whitney, or *t*-test with Welch correction, followed by F-test to compare variances), repeated-measures ANOVA followed by multiple-comparison Newman-Keuls post-test, Kruskal-Wallis test (with Dunn’s multiple comparison test), one-way ANOVA followed by Bartlett’s test for equal variances, linear regression analysis, Fisher’s exact test and Chi-square test were carried out. The significance cut-off was set at *p* < 0.05.

## 3. Results

Data are reported throughout as average ± SEM unless otherwise stated.

Patients were 83 females and 53 males. Their mean age was 63.6 ± 1.09 year.

**Figure 1:**
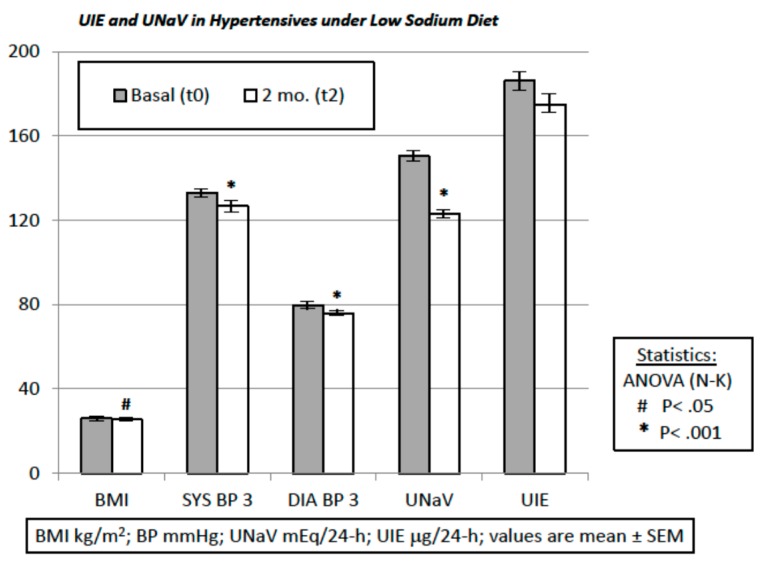
Main Data. *n* = 136 pts, 83 females and 53 males. ANOVA followed by Newman-Keuls post-test for repeated measures. Data are reported as mean and SD.

**Figure 2:**
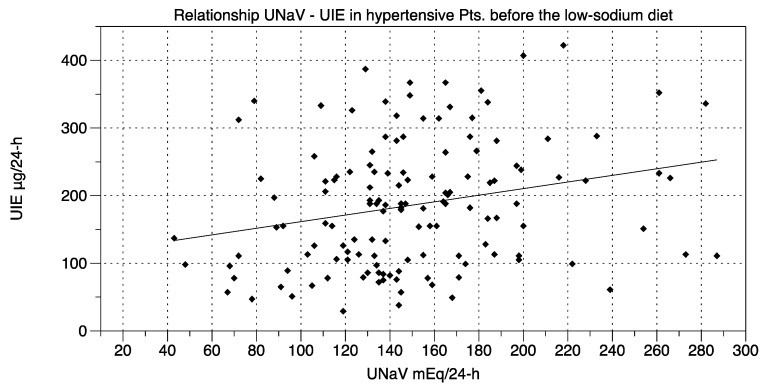
Relationship between UNaV (mEq/24-h) and UIE (µg/24-h) before the diet period (time t0). Statistics: *r* = 0.246; *p* = 0.004.

**Figure 3:**
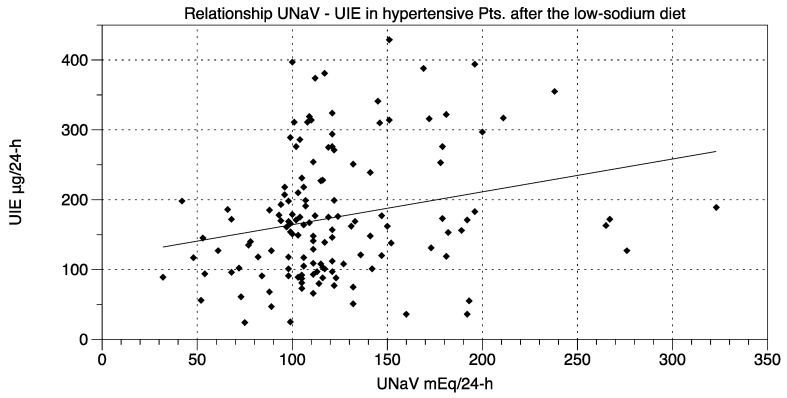
Relationship between UNaV (mEq/24-h) and UIE (µg/24-h) after the diet period (time t2). Statistics: *r* = 0.238; *p* = 0.005.

Our intervention consisted of proposing a simple low-sodium protocol. The time-span between the administration of the dietary protocol (visit at time t0) and the final visit (time t2) was 9 ± 0.12 weeks:

- BMI was 25.86 ± 0.40 kg/m^2^ before the dietary suggestions (visit t0) and 25.38 ± 0.37 kg/m^2^ afterwards (visit t2) (*t*-test and repeated-measures ANOVA and Newman-Keuls post-test, *p* < 0.05).
- UNaV decreased from 150.3 ± 4.01 mEq/24-h at visit t0 to 122.8 ± 3.92 mEq/24-h at visit t2 (repeated-measures ANOVA and post-test, *p* < 0.001).
- Both systolic and diastolic BP values decreased significantly from visit t0 to visit t2 (by 6.15 ± 1.32 mmHg and by 3.75 ± 0.94 mmHg, respectively. ANOVA and post-test, *p* < 0.001) (Figure 1).
- Drug consumption also decreased from visit t0 to visit t2 (ΔDDD 0.29 ± 0.06, *t*-test, *p* < 0.05).
- Median UIE global values were 184.2 µg/24-h (lower and upper 95% CI 170.3 and 201.9) at t0, and 162.0 µg/24-h (lower and upper 95% CI 159.6 and 190.3) at t2.
- Median UIE values in females were 178.2 µg/24-h (lower and upper 95% CI 163.0 and 204.3) at t0, and 153.7 µg/24-h (lower and upper 95% CI 151.3 and 188.5) at t2.
- Median UIE values in males were 188.0 µg/24-h (lower and upper 95% CI 164.9 and 215.2) at t0, and 170.0 µg/24-h (lower and upper 95% CI 155.9 and 210.5) at t2. One way ANOVA (F = 0.6105, *R* square 0.006788) showed non-significant differences between UIE values in males vs. females both before and after the diet period (*p* = 0.6087). Bartlett’s test for equal variances gave non-significant results (Bartlett’s statistics 1.449, *p* = 0.6940, Newman-Keuls multiple comparison test *p* > 0.05).
- UIE was below 100 µg/24-h in 28 patients before the suggested diet, and in 28 patients thereafter (Fisher’s exact test: *p* = NS; Chi-square, df: 0.0, 1; *p* = NS).
- UIE decreased from 186.1 ± 7.95 µg/24-h at visit t0, to 175.0 ± 7.74 µg/24-h at visit t2 (repeated-measures ANOVA and post-test, *p* = NS). Furthermore, these data were reanalyzed with non-parametric tests (Mann-Whitney, or Welch correction, *t* = 1.002, df = 269, 95% C.I. −10.67 to +32.97), to avoid distributional assumptions (again, differences were non-significant: *p* = 0.2737 to *p* = 0.3173). A possible UIE variability induced by the dietary suggestions was challenged with an F test to compare variances before and after the observation period. F test showed non-significant differences (F, DFn, Dfd, 1057, 135, 135; *p* = 0.7484).
- Significant relationships were found between UNaV and UI both before the administration of the protocol, at visit t0 (*r* = 0.246, *p* = 0.004) (Figure 2) and after, at visit t2 (*r* = 0.238, *p* = 0.005) (Figure 3).
- UNaV and UIE were significantly associated both before and after the dietary suggestions (Chi-square test; df at t0 75.55, 1; df at t2 205.6, 1; *p* < 0.0001 for both).

## 4. Discussion

The proposed low-sodium diet induced a significant reduction in UNaV, BMI, BP, and DDD in our patients. Urinary iodine excretion showed a small, non-significant decrease.

We did not monitor the actual diet in our patients, because of the inherent limits of FFQ and 24-h recall methods [19]. Instead, we relied on the UNaV, which is usually considered the gold standard for the assessment of the sodium content of a diet [19]. Similarly, we relied on the UIE as a reference index of iodine intake [20,21].

In our patients we did not evaluate urinary creatinine excretion, which is of limited value as a measure of the completeness of 24-h collection [32,33], because of the high variability of creatinine in urine, with a reported SD as high as 29 to 79% of the mean [32,34], with sensitivity and specificity for identifying incomplete collections as low as 6% and 57%, respectively [35]. Instead, we relied on the total 24-h urine volumes, with a high cut-off of 700 mL [30].

In our patients, the decrease in UNaV after the dietary suggestions was paralleled by significant improvements in BMI and BP values, with an additional significant decrease in drug consumption.

Our patients seemed to follow our suggestions, apparently reducing their sodium intake from (150.3 mEq × 23 =) 3.46 g/day, which is equivalent to 8.78 g/day of salt, to (122.8 mEq × 23 =) 2.82 g/day, which is equivalent to 7.17 g/day of salt. This small but significant decrease, albeit lower than that suggested in the Guidelines [1,2,3,4], yielded a significant improvement in our patients’ BP values (even more important in light of their reduced drug intake), once again displaying the favorable effect of a low-sodium approach to arterial pressure control.

This effective UNaV reduction did not appear to affect UIE, which showed a non-significant reduction from 186.1 to 175.0 µg/24-h. The approximate iodine intake thus decreased from (186.1/0.92 =) 202.3 μg/day, to (175.0/0.92 =) 190.2 μg/day [25,36].

The number of iodine-deficient patients (UIE below 100 µg/24-h) did not change from the basal observation to the final visit (28/136 to 28/136, i.e., 20.59%).

Our data agree with previously published findings, which indicate that reducing sodium consumption in an iodine-sufficient population does not compromise iodine intake [26]. In our hypertensive patients, a successful intervention (in the form of dietary salt intake suggestions) was followed by favorable effects in terms of UNaV, BMI, and BP reduction (even with a reduction in the need for drugs).

Our instructions do not appear to induce a substantial nor significant decrease in iodine intake, and the number of iodine-deficient subjects did not change substantially, nor significantly (actually, it did not change at all). Differences between females and males were non-significant.

In Italy, the primary source of salt is not processed or canned food (the use of which remains negligible), nor the amount of salt added at the table or during cooking; rather, it is constituted by three categories of food: bread, cheese, and cured meats [37]. The aim of our dietary suggestions was to reduce sodium intake, while minimizing the iodine decrease. On our market, sales of iodized table salt have risen to a current level of the 60% [15]. Although our district, the Liguria Region, has recently achieved iodine sufficiency, Italy (as noted in the Introduction) suffers from mild iodine deficiency [29]. This situation is reflected in our data, which showed average iodine sufficiency (average UIE 186.1 µg/24-h, median UIE 184.2 µg/24-h; lower and upper 95% CI 170.3 and 201.9 before the dietary suggestions), but with 20% of iodine-deficient subjects (UIE < 100 µg/24-h). Efforts to reduce salt intake in our country must take this fragile IS into account. This is why our suggestions were aimed at reducing the main sources of salt (bread, cheese, and cured meats), whereas table salt was not banned; we therefore managed not to cut a possible main source of iodine.

The fact that UNaV and UIE were significantly related and associated, both before and after the administration of the diet protocol, suggests a possible common source of both sodium and iodine; given that the three salty foods had been excluded from the dietary protocol, this common source was presumably table salt. Our results support this view: the non-significant decrease in UIE seen in our patients seems to fit in with the current low use of iodized salt (3–8%) by the Italian food industry [28], with the implication that table salt remains the main source of iodine in our country [28].

## 5. Conclusions

In an area of iodine sufficiency, albeit in a mild iodine-deficient country, where table salt is the main source of iodine, a low-sodium approach which primarily limits the salty foods (instead of a “no salt added” suggestion) seems safe in terms of iodine intake, and does not worsen the IS.
